# Rethinking Invasive Mediastinal Staging in the Era of Neoadjuvant Immune‐Checkpoint Inhibitors

**DOI:** 10.1111/1759-7714.70292

**Published:** 2026-05-01

**Authors:** Saulo Brito Silva, Danilo Tadao Wada, Lycio Umeda Dessotte, Li Siyuan Wada, Adilson Aparecido Faccio, Federico Garcia Cipriano

**Affiliations:** ^1^ Department of Medical Imaging, Hematology and Oncology, Ribeirão Preto Medical School University of São Paulo Ribeirão Preto Brazil; ^2^ Department of Surgery and Anatomy, Ribeirão Preto Medical School University of São Paulo Ribeirão Preto Brazil; ^3^ Department of Internal Medicine, Ribeirão Preto Medical School University of São Paulo Ribeirão Preto Brazil

**Keywords:** immune checkpoint inhibitors, mediastinal staging, neoadjuvant immunotherapy, nonsmall cell lung cancer

## Abstract

Over the past several decades, the management of nonmetastatic nonsmall cell lung cancer (NSCLC) has centered on identifying patients eligible for upfront surgical treatment. This selection has traditionally relied on multidisciplinary assessments of clinical operability and oncologic resectability, with the latter depending primarily on mediastinal lymph node evaluation, a key prognostic factor in determining whether patients should be directed toward upfront surgery or definitive chemoradiotherapy. The recent incorporation of immune checkpoint inhibitors (ICIs) into standard neoadjuvant therapy has transformed this paradigm. By significantly enhancing pathologic response and improving survival across the full spectrum of N2 disease, neoadjuvant ICI therapy is reshaping the prognostic weight traditionally assigned to mediastinal nodal involvement, challenging long‐standing staging practices. Rather than serving primarily to exclude patients from surgery, mediastinal assessment in the immunotherapy era may play a more selective role in baseline risk stratification while also potentially gaining new roles, such as in the evaluation of treatment response and nodal downstaging, which could support broader refinements in clinical decision‐making. This update synthesizes emerging evidence and evolving clinical concepts to re‐examine mediastinal assessment in the immunotherapy era, with implications for clinical decision‐making and future trial design in nonmetastatic NSCLC.

## Graphical abstract

As neoadjuvant immunotherapy reshapes resectability in NSCLC, the role of invasive mediastinal staging is being fundamentally reconsidered—with implications for risk stratification, patient selection, and trial design.

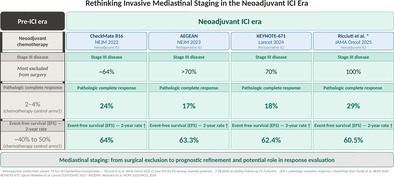

## 1 Introduction

Lung cancer remains the world's deadliest malignancy, responsible for an estimated 1.82 million deaths in 2022 [1]. Over the past decade, population‐level mortality has declined, reflecting not only advances in systemic therapy for metastatic disease but also a gradual shift toward earlier‐stage diagnoses, driven largely by the broader adoption of lung imaging and screening programs [2]. Although survival among patients with metastatic nonsmall cell lung cancer (NSCLC) has improved substantially, they still face an inevitably incurable trajectory [3]. As such, the prospect of cure continues to rely fundamentally on optimizing the management of nonmetastatic disease, for which surgical resection remains the principal curative approach in appropriately selected patients [4].

Patients with nonmetastatic NSCLC and either no nodal involvement or ipsilateral hilar disease are routinely considered operative candidates. In contrast, those with ipsilateral mediastinal nodal involvement (N2 disease) have traditionally occupied a therapeutic gray zone, largely because the probability of cure after surgery rarely exceeds 20%–40% across most reported surgical series [5, 6, 7]. Indeed, radiologic N2 involvement is strongly associated with micrometastatic dissemination at the time of presentation, contributing to high rates of early systemic relapse, typically within 6 to 15 months after resection [8, 9]. Taken together, these factors have historically underpinned the poor outcomes in this population and fueled a progressive reluctance to pursue surgery as a standard therapeutic approach.

The publication of the PACIFIC Phase III trial further reinforced a nonsurgical paradigm for many Stage III presentations, demonstrating a meaningful survival benefit with 12 months of durvalumab after definitive chemoradiotherapy [10]. Yet, despite this advance, 77% of patients eventually relapsed in the trial, underscoring persistent unmet needs in the curative‐intent management of Stage III disease [11]. Importantly, more than half of the PACIFIC trial population consisted of Stage IIIA patients according to the AJCC 6th edition. As such, a substantial proportion of individuals enrolled in PACIFIC might—under contemporary multidisciplinary evaluation—still be considered potential candidates for surgery.

It is essential to recognize that clinical N2 disease encompasses a broad spectrum of prognostic scenarios. Adverse features such as bulky nodes (≥ 2.5 cm short axis), radiologic indicators of extranodal extension (e.g., encasement or invasion of mediastinal vessels or airways), and multistation involvement are strongly associated with worse outcomes [12]. This heterogeneity motivated the 9th edition of the AJCC staging system to incorporate part of this anatomically defined prognostic spectrum by subdividing N2 disease into N2a (single‐station) and N2b (multistation) categories [13].

However, additional prognostic determinants beyond imaging likely influence outcomes and may merit consideration in future AJCC iterations, paralleling refinements already observed in other malignancies such as head and neck or breast cancer [14, 15]. For example, molecular and biological tumor profiles—particularly EGFR‐ or ALK‐driven NSCLC—may confer a distinctly heightened propensity for early systemic and CNS dissemination, introducing prognostic dimensions that can directly shape therapeutic decision‐making, even when the disease appears anatomically operable [16].

## 2 Preoperative Workup and Resectability Criteria in NSCLC

Clinical operability in nonsmall cell lung cancer (NSCLC) refers to a patient's physiological capacity to tolerate lung resection and its cardiopulmonary demands. It requires an integrated assessment of comorbidities, pulmonary and cardiac reserve, functional status, and overall frailty—factors that carry particular weight in a population with a high burden of smoking‐related impairment [17].

Preoperative assessment follows a structured algorithm beginning with spirometry and diffusing capacity, followed by estimation of predicted postoperative (ppo) values using anatomical segment counting. This method is reliable when parenchymal function is relatively homogeneous [17]. However, when disease distribution is heterogeneous—as in emphysema, impaired regional perfusion, or airway obstruction—functional imaging becomes essential. Ventilation–perfusion scintigraphy refines regional estimates, while SPECT/CT provides three‐dimensional quantification that more accurately correlates with observed postoperative FEV_1_ and DLCO, often reclassifying borderline candidates by identifying hypofunctional regions whose removal would minimally affect global pulmonary reserve [18, 19]. Current guidelines define ppoFEV_1_ and ppoDLCO ≥ 40% predicted as acceptable thresholds for major resection, whereas lower values warrant cardiopulmonary exercise testing to quantify integrated physiological capacity. A VO_2_max < 10 mL/kg/min (or < 35% predicted) denotes prohibitive operative risk, while values > 15–20 mL/kg/min correlate with favorable postoperative outcomes. Measures such as the VE/VCO_2_ slope may further refine risk estimation, as patients with elevated VE/VCO_2_ (> 35–40) are considered at high risk for postoperative complications and mortality [20, 21, 22].

Oncologic resectability, in contrast, encompasses more than the technical feasibility of achieving an R0 resection with negative bronchial, vascular, and pleural margins. It also requires comprehensive mediastinal and hilar lymph‐node dissection to ensure accurate pathologic staging [23]. The concept further incorporates the estimated risk of early systemic relapse after surgery, which influences decision‐making in Stage III disease [24]. From a nodal standpoint, N3 involvement—contralateral mediastinal or supraclavicular lymph nodes—constitutes an absolute criterion for unresectability, whereas clinical N2 disease remains a heterogeneous category requiring individualized assessment that accounts for the number of involved stations, the pattern of spread, and the presence of bulky or multistation disease [25]. According to IASLC criteria, a resection is considered complete only when margins are microscopically negative, extracapsular extension is absent, and at least three mediastinal nodal stations have been systematically removed [26].

Mediastinal staging typically begins with contrast‐enhanced CT and PET‐CT [27]. PET is the most accurate noninvasive modality for mediastinal nodal assessment in NSCLC, with pooled analyses showing sensitivity near 80% and specificity approaching 90%, implying lower false‐negative than false‐positive rates [28, 29]. However, its performance varies substantially across clinical scenarios. Histologic subtypes such as nonsquamous or micropapillary adenocarcinoma, central tumor location, radiographic N1 involvement, and a high mediastinal‐to‐primary SUVmax ratio are all associated with a higher likelihood of false‐negative PET findings [29, 30].

Given the concern about inadvertently proceeding to surgery in the presence of occult mediastinal metastases, and considering the historical context in which such patients had poor outcomes with surgery compared with definitive chemoradiation, refining mediastinal evaluation to exclude false‐negatives has become standard practice in patients with a high pretest probability of N2/N3 disease. These assessments can be performed using minimally invasive procedures, namely endoscopic ultrasound‐guided fine‐needle aspiration (EUS) and endobronchial ultrasound‐guided transbronchial needle aspiration (EBUS), or surgical approaches such as mediastinoscopy. Mediastinoscopy, performed via a cervical approach under general anesthesia, has the advantage of reliably accessing paratracheal and anterior subcarinal stations, as well as prevascular (3A) and pretracheal (3P) nodes that cannot be consistently sampled by EBUS or EUS. Of note, only patients with small peripheral stage IA tumors and no nodal abnormalities on cross‐sectional or metabolic imaging are exempted from invasive sampling, due to the low likelihood of clinically occult mediastinal metastases [30].

## 3 Revisiting the Paradigm of Operable N2 NSCLC in the Era of Neoadjuvant Immune‐Checkpoint Inhibitors

Historically, neoadjuvant chemotherapy has not been considered standard of care in operable NSCLC. Its absolute survival benefit has been modest—approximately 5%—and comparable to that of adjuvant therapy, but with the drawback of virtually negligible rates of meaningful pathologic response [31, 32]. Consequently, and further influenced by concerns that disease progression during neoadjuvant treatment could preclude surgical resection, upfront surgery followed by adjuvant chemotherapy remained the dominant approach. In practice, neoadjuvant chemotherapy was reserved largely for patients deemed borderline resectable, serving primarily as a biological stress test to refine surgical candidacy [32].

Nevertheless, the advent of neoadjuvant immune checkpoint inhibitors (ICIs) has dramatically reshaped this landscape. Incorporating ICIs into the neoadjuvant treatment of NSCLC represents one of the most significant therapeutic advances in the curative setting of lung cancer to date [33]. The biological rationale is compelling: administering immunotherapy while the tumor and its microenvironment remain intact maximizes antigen presentation, enhances T‐cell priming, and may elicit more robust and durable antitumor immunity [34]. Early pioneering studies demonstrated that as few as two preoperative doses of nivolumab yielded major pathologic response (MPR) in 45% of patients and pathologic complete response (pCR) in 15% [35]. Subsequently, Phase II and III clinical trials evaluating chemoimmunotherapy combinations not only validated these findings but reported impressive pCR rates of 17%–40%, with favorable safety profiles and no evidence of impaired surgical feasibility or resectability [33]. Importantly, long‐term follow‐up from CheckMate 816 confirmed sustained clinical benefit, including a 10% absolute improvement in overall survival with three cycles of neoadjuvant nivolumab plus chemotherapy [36].

Remarkably, a substantial proportion of patients enrolled in phase III neoadjuvant and perioperative trials had mediastinal nodal involvement. Across the CheckMate, AEGEAN, and KEYNOTE programs, 59%–63% of participants presented with N2 disease [37, 38, 39]. Nonetheless, these studies did not classify nodal disease according to the AJCC 9th edition (i.e., N2a vs. N2b) and provided limited detail on the number of involved nodal stations, the presence of bulky or invasive nodes, or the extent of preoperative invasive staging. Notwithstanding, available subgroup analyses suggest that adding ICIs confers similar and perhaps even greater benefit among such prognostically unfavorable patients, particularly those with multistation N2 disease [40, 41]. Indeed, a recent meta‐analysis reported a pooled hazard ratio for event‐free survival of 0.50 (95% CI, 0.32–0.78) specifically for tumors with multistation N2 involvement, underscoring that these biologically aggressive presentations may derive substantial long‐term benefit from neoadjuvant immunotherapy [42].

Further reinforcing this evolving paradigm, emerging evidence from real‐world populations with poor‐prognostic nodal features considered borderline or unresectable (T4 and/or N2 or N3 disease) adds compelling support. In a paradigm‐shifting multicenter cohort treated with pembrolizumab, nivolumab, or durvalumab combined with platinum‐based chemotherapy for three to four cycles, 69.6% of patients had multistation N2 disease and 9.8% had N3 involvement. Despite this high‐risk profile, patients achieved meaningful pathological responses, with pCR and MPR rates of 29% and 42%, respectively, and a median event‐free survival of 52.6 months (95% CI, 27.8 to not reached) [43]. Altogether, these findings underscore how neoadjuvant immunotherapy is redefining long‐standing assumptions and expanding the therapeutic horizon for patients historically excluded from trials of resectable NSCLC.

Thus, the traditional rationale for invasive mediastinal staging warrants reinterpretation in the era of neoadjuvant chemoimmunotherapy. Historically, the identification of N2–N3 disease functioned largely as a determinant of surgical eligibility. Today, however, many patients across selected mediastinal subsets derive meaningful benefit from neoadjuvant regimens and may still be considered for resection, shifting the focus from “operability exclusion” to “prognostic and therapeutic refinement,” while ensuring that patients with unequivocal indications for neoadjuvant therapy can initiate treatment promptly to maximize the curative potential.

This reappraisal also highlights the importance of addressing logistical and technical barriers that may limit timely access to invasive mediastinal staging. Access to EBUS, EUS, and mediastinoscopy remains uneven across many healthcare systems, particularly in low‐ and middle‐income countries with limited resources, trained personnel, or operating room availability. From a practical standpoint, performing invasive mediastinal staging in patients already indicated for neoadjuvant ICIs requires careful coordination to avoid delays in initiating systemic therapy, which is critical for maximizing both the biological and curative benefits of treatment. Potential delays may arise from scheduling bottlenecks, surgical waitlists, or postprocedural recovery, which can be exacerbated by procedure‐related complications. Although complications are relatively uncommon, they are not negligible—particularly with mediastinoscopy—and real‐world rates may be higher in centers still early in their learning curves [44, 45].

Another key consideration is the intrinsic limitation of conventional histopathologic assessment in accurately detecting mediastinal micrometastases. The sensitivity of endosonographic biopsy for radiographically normal mediastinal lymph nodes remains suboptimal at approximately 49%, largely because most unforeseen metastatic deposits are micrometastatic, typically measuring less than 3–5 mm [46, 47]. Moreover, when EBUS/EUS is complemented with mediastinoscopy, approximately 20% of patients initially staged as radiographic N0 ultimately prove to harbor pN2 metastases, as demonstrated in the meta‐analysis by Leong et al., highlighting the inherent unpredictability of pathological sampling for microscopic disease [48]. These diagnostic limitations suggest that detecting micrometastatic N2 disease may have limited clinical impact in patients already indicated for neoadjuvant therapy, particularly those with tumors > 4 cm or N1 involvement but no radiographically suspicious mediastinal nodes. In these scenarios, the presence of occult micrometastatic mediastinal disease would neither alter therapeutic decisions nor meaningfully affect outcomes once neoadjuvant chemoimmunotherapy is initiated. Supporting this concept, evidence from other malignancies shows that micrometastatic nodal disease behaves differently from macrometastatic spread; for example, in breast cancer, sentinel‐node micrometastases are associated with low rates of further nodal involvement and generally favorable outcomes [49].

With respect to ruling out occult N3 involvement, available data consistently show its prevalence to be low. Riquet et al. reported that among 586 patients with pathologic N2 disease—66% single‐station and 34% multistation—only 2% harbored occult N3 metastases upon systematic lymphadenectomy, a finding mirrored by Casali et al., who observed unexpected N3 in fewer than 2% of 183 patients with pathologic N2 [50, 51]. Prospective evidence reinforces this pattern: in the multicenter SEISMIC study, unsuspected N3 detected via systematic EBUS/EUS occurred in no more than 7% of patients with cN0–N2 disease, with similar rates within the cN2 subgroup [52]. Given the low overall prevalence, identifying which patients are at higher risk for occult N3 involvement remains an important, yet incompletely understood, question. Sakao et al., studying upper‐lobe tumors with clinical N2 defined by enlarged mediastinal nodes on CT, found that involvement of multiple mediastinal stations and higher‐level nodal spread were associated with unexpected cervical or contralateral mediastinal metastases [53]. In the absence of more definitive evidence, current expert recommendations—including those from the Society of Thoracic Surgeons—remain largely inferential and suggest that bulky or multistation N2 may indicate a higher risk of N3 involvement [54]. Most importantly, the more consequential question is how the detection of occult N3 involvement should influence modern treatment algorithms, particularly when neoadjuvant immunotherapy may mitigate its adverse prognostic impact, as illustrated by the study by Ricciuti et al., in which 9.8% of the cohort had N3 disease yet still achieved substantial pathologic and survival benefit [43].

## 4 Implications for Clinical Practice and Future Trials

We propose a risk‐adapted workflow to be discussed within multidisciplinary tumor boards, aiming to provide personalized mediastinal assessment for patients undergoing neoadjuvant chemoimmunotherapy. This model is intended to support flexible, evidence‐based decisions that fit the current era of neoadjuvant immunotherapy, facilitating timely initiation of treatment and maximizing its curative potential. By offering structured guidance, it may also help harmonize inclusion criteria in future studies and provide safe and efficient pathways in real‐world practice.

Within this updated framework, invasive mediastinal staging remains essential for patients without clear radiologic indications for neoadjuvant therapy, as pathologic confirmation directly determines whether induction treatment is indicated. In patients already deemed candidates for neoadjuvant chemoimmunotherapy based on tumor size (> 4 cm) or radiographic N1 involvement with no mediastinal radiologic alteration, the impact of invasive staging on the initial treatment decision is more limited, although it can still clarify indeterminate findings. In practical terms, real‐world evidence shows that a substantial proportion of such patients—up to 40%, according to the STS database analysis by Krantz et al. [55]—undergo invasive mediastinal assessment in routine practice, highlighting the potential value of a risk‐adapted workflow to optimize procedural planning and resource allocation.

**FIGURE 1:**
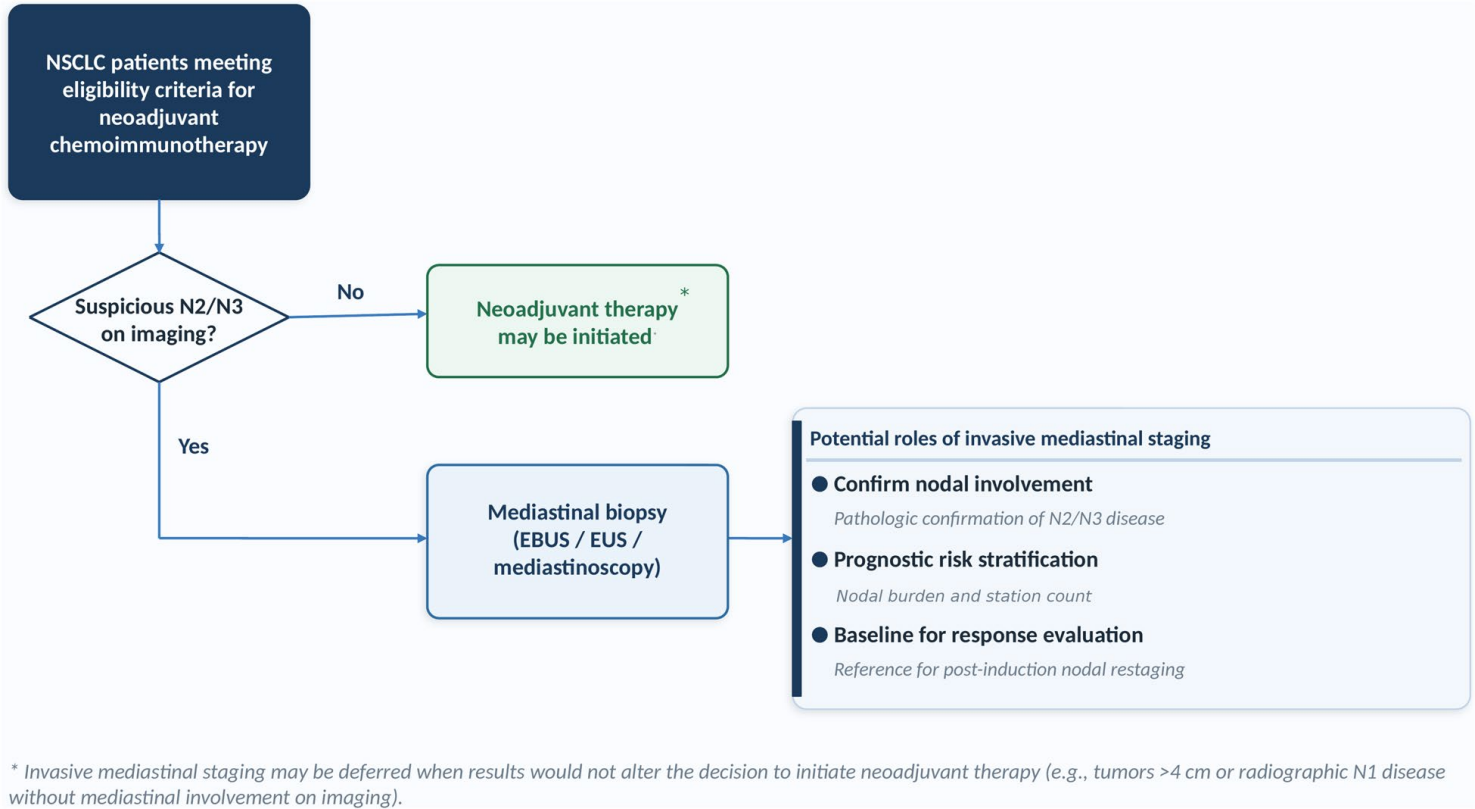
Proposed decision framework for invasive mediastinal staging in patients with nonsmall cell lung cancer being considered for neoadjuvant chemoimmunotherapy. When imaging raises no suspicion of N2/N3 nodal involvement, neoadjuvant therapy may be initiated without prior tissue sampling. When mediastinal involvement is suspected, biopsy via EBUS, EUS, or mediastinoscopy is recommended to confirm nodal disease, stratify prognostic risk, and establish a baseline for postinduction response evaluation.

In patients with suspicious mediastinal nodes on imaging who are candidates for neoadjuvant chemoimmunotherapy, invasive mediastinal staging is recommended to confirm nodal involvement, perform accurate prognostic risk stratification—including assessment of nodal burden and station count—and potentially establish a baseline reference for evaluating nodal response after induction therapy. This baseline may be particularly valuable for designing future trials aimed at refining curative‐intent strategies, including the choice between surgery and definitive chemoradiotherapy. The potential roles of invasive mediastinal staging in this setting are illustrated in Figure 1.

## Author Contributions

**Saulo Brito Silva:** conceptualization, methodology, literature search and synthesis, writing – original draft, writing – review and editing, visualization. **Danilo Tadao Wada:** methodology, writing – review and editing. **Lycio Umeda Dessotte:** methodology, writing – review and editing. **Li Siyuan Wada:** methodology, writing – review and editing. **Adilson Aparecido Faccio:** methodology, writing – review and editing. **Federico Garcia Cipriano:** conceptualization, supervision, project administration, writing – review and editing.

## Funding

The authors have nothing to report.

## Ethics Statement

This manuscript is a narrative review and did not involve human participants, animal experiments, or collection of personal data. Ethical approval was therefore not required.

## Consent

This manuscript is a narrative review and does not involve human participants or personal data.

## Conflicts of Interest

The authors declare no conflicts of interest.

## Data Availability

Data sharing is not applicable to this article as no datasets were generated or analysed during the current study.
